# Optimization of Chitosan Synthesis Process Parameters to Enhance PES/Chitosan Membrane Performance for the Treatment of Acid Mine Drainage (AMD)

**DOI:** 10.3390/ma17112562

**Published:** 2024-05-26

**Authors:** Ndiwanga F. Rasifudi, Lukhanyo Mekuto, Machodi J. Mathaba

**Affiliations:** 1 Department of Chemical Engineering, University of Johannesburg, P.O. Box 17011, Doornfontein 2028, South Africa; nfrasifudi@gmail.com; 2 Institute for Catalysis and Energy Solutions, University of South Africa, Private Bag X6, Florida 1710, South Africa; mathamj@unisa.ac.za

**Keywords:** acid mine drainage (AMD), chitosan, deacetylation, hydrophilicity, PES, membrane

## Abstract

Acid mine drainage (AMD) is an environmental issue linked with mining activities, causing the release of toxic water from mining areas. Polyethersulphone (PES) membranes are explored for AMD treatment, but their limited hydrophilicity hinders their performance. Chitosan enhances hydrophilicity, addressing this issue. However, the effectiveness depends on chitosan’s degree of deacetylation (DD), determined during the deacetylation process for chitosan production. This study optimized the chitin deacetylation temperature, alkaline (NaOH) concentration, and reaction time, yielding the highest chitosan degree of deacetylation (DD) for PES/chitosan membrane applications. Prior research has shown that high DD chitosan enhances membrane antifouling and hydrophilicity, increasing contaminant rejection and permeate flux. Evaluation of the best deacetylation conditions in terms of temperature (80, 100, 120 °C), NaOH concentration (20, 40, 60 wt.%), and time (2, 4, 6 h) was performed. The highest chitosan DD obtained was 87.11% at 80 °C, 40 wt. %NaOH at 4 h of chitin deacetylation. The PES/0.75 chitosan membrane (87.11%DD) showed an increase in surface hydrophilicity (63.62° contact angle) as compared to the pristine PES membrane (72.83° contact angle). This was an indicated improvement in membrane performance. Thus, presumably leading to high contaminant rejection and permeate flux in the AMD treatment context, postulate to literature.

## 1. Introduction

Acid mine drainage (AMD) is an environmental issue linked with mining activities and operations, and it is the flow of toxic, polluted water from mining areas. AMD carries metals, radionuclides (radiation emitting atoms), and salts in hazardous concentrations to human life, animal life, and the environment [1]. AMD is a significant water quality problem as it reduces the pH of water resources, making dissolved metals readily available to fish and benthic organisms, which becomes a major pathway of their introduction into the human food chain [2]. Acid mine drainage affects an estimated 22,000 km of streams and 180,000 acres of freshwater lakes in the United States [3]. Significant AMD impact on Australia’s water bodies has also been reported on over 13,000 km of streams [4]. Moreover, approximately 9500 km of waterways have been affected in Canada [5]. In South Africa, the AMD problem is chronic due to the facilities being already exhausted by the current water treatment challenges. When sulfide-bearing material is exposed to oxygen and water, acid mine drainage (AMD) occurs [3]. AMD is most commonly, but not always, produced in iron sulfide-aggregated rocks. Although this process happens naturally, mining activities hasten the occurrence of AMD by increasing the number of sulfides exposed via tailing dumps or abandoned mine overflow [6].

Due to water quality requirements and environmental concerns, membrane technology has gained significant attention for application in acid water treatment. It has proven to be a promising alternative to traditional processes due to its low cost and separation efficiency [7]. However, during operations, Polyethersulphone (PES) membranes are limited to operating at their full performance due to fouling and their relatively hydrophobic nature. Numerous studies [7,8] have revealed that infusing chitosan in PES membranes improves its antifouling properties and hydrophilicity. The hydrophobic nature of PES membranes caused by the sulfonyl group linking the phenylene rings leads to the accumulation and deposition of material on the membrane’s surface, leading to low contaminant rejection. Chitosan has been identified as a hydrophilicity-enhancing agent that can be infused in PES membranes to improve its performance [9]. The extent to which chitosan poses this positive effect is dependent upon chitosan’s degree of deacetylation (DD), the extent to which the acetyl group has been removed from the polymer chain. Currently, commercial chitosan is available with a DD of about 80%. It has been established that chitosan of high DD means greater improvement in membrane performance. The objectives of this study were to establish the optimum temperature, alkaline (NaOH) concentration, and reaction time that yields the highest chitosan DD, to characterize the synthesized PES/chitosan membrane synthesized with obtained chitosan of the highest DD, and to examine the impact of chitosan degree of deacetylation on the quality and separation efficiency (cation and anion binding) of the chitosan-infused PES membrane.

Chitosan is obtained from chitin through the deacetylation process [10]. Chitin precedes in crustacean shells such as crabs, shrimps, and prawns, which can also be obtained from seafood industry waste; hence, the study also holds a waste beneficiation narrative. The deacetylation process involves the removal of acetyl groups from the molecular chain of chitin, hence deacetylation, leaving behind complete amino groups (–NH_2_) [10]. When chitosan is used to modify polymeric membranes for acid mine drainage (AMD) treatment, it is expected that a large number of amino groups available should translate into a more effective sorption capacity [8]. In this study, chitosan synthesis was optimized at different temperatures, NaOH concentrations, and reaction times to yield different values of DD, which were then studied to understand the trends and conclude the optimum conditions. Then, chitosan synthesized at optimum conditions was used to fabricate a PES/chitosan membrane whose performance-indicating parameters were evaluated against a pristine PES membrane. Research has explored the incorporation of high-DD chitosan into PES membranes. However, the currently commercialized synthesis process, involving high temperatures exceeding 100 °C and elevated concentrations of NaOH, undermines its viability [11].

This study addresses these limitations by establishing chitosan synthesis conditions that are economically viable, sustainable, and feasible, emphasizing the importance of lower NaOH concentrations and temperatures for improved process efficiency. The study pursued optimizing chitosan’s degree of deacetylation by drawing upon established findings from prior research. The experimental approach centered on selecting key variable ranges well-documented in the literature and was inspired by the Box–Behnken design (BBD) of experiments. Specifically, the study focused on a temperature range spanning 80–120 °C, NaOH concentrations within the 20–60% range, and reaction times of 2–6 h. This approach leveraged the extensive existing knowledge, thereby enabling the confirmation and refinement of these previously identified optimization parameters. Additionally, the authors sought to uncover the critical points of interdependencies among these factors. The decision to forego the use of a formal experimental design, such as a Central Composite Design (CCD), was a deliberate choice driven by the aim to obtain the highest DD around the area of highest response while also accessing the interdependencies.

The role of membrane technology in treating acid mine drainage (AMD) is crucial, with membrane properties such as surface charge, hydrophilicity, and fouling propensity being significantly influenced by ionic strength and pH [12]. An increase in ionic strength can alter electrostatic interactions, affecting fouling behavior, while pH can impact membrane surface charge and energy, influencing fouling and separation performance [12]. The use of negatively charged membranes in dyeing wastewater treatment has been found to enhance antifouling properties and increase flux [13]. However, the inclusion of chitosan in the membrane matrix can mitigate these effects. Chitosan, with its positively charged amino groups, can electrostatically interact with negatively charged contaminants, enhancing fouling resistance. Moreover, chitosan modification can improve membrane hydrophilicity, thus reducing fouling propensity [14]. Nevertheless, membrane fouling, a common issue in membrane distillation, can lead to a reduction in flux, with the vapor-pressure depression being a dominant cause [15]. To address this, nanocomposite membranes with hydrophilic and antifouling properties have been developed [16]. These studies collectively underscore the importance of considering ionic strength and pH in membrane performance, particularly in the context of AMD treatment.

## 2. Experimental Set-Up

### 2.1. Materials and Chemicals

The chitosan was synthesized from shrimp shells obtained from Ocean Basket restaurant, Eastgate, South Africa. Chemicals such as sodium hydroxide (NaOH), PES granules, and solvent dimethyl sulfoxide (DMSO) were purchased from Rochelle Chemicals, a chemicals supplier (Johannesburg, South Africa). The purchased chemicals were of analytical grade; thus, no further purification was required.

### 2.2. Equipment

A 200 µm sieve was used to sieve the crushed and milled shrimp cells to ensure that at least 75 wt.% was less than 200 µm, this is a significant step in protein recovery and wastewater treatment applications [17,18]. A laser diffraction Particle size distribution (PSD) Malvern analyzer (Malvern Pananalytical, England, UK) was used to analyze the raw shell’s PSD to validate the particle size. Fourier Transform Infrared (FTIR) (ThermoFisher Scientific, Waltham, MA, USA) was used to characterize the raw shell and the chitosan produced thereof. FTIR spectroscopy was used to identify the functional groups in the chitosan and synthesized PES/chitosan membrane. Its adsorption ratios, A1320 and A1340 were used for the determination of the degree of deacetylation. A scanning electron microscope (SEM) was used for surface imaging of the membranes. A contact angle analyzer (DataPhysics Instruments, Charlotte, NC, USA) was used to measure the water contact angle of the fabricated pristine PES and PES/chitosan membranes.

### 2.3. Production of Chitosan

The following steps were carried out sequentially to synthesize chitosan from the milled chitin. The below methodology was essentially adapted from [7].

Step 1: Pulverization of shells

Shrimp shells were first boiled and dried as a measure to remove all the impurities before crushing and using them. This step was followed by sieving (200 µm) to ensure that at least 75% of the total mass exhibited a particle size of less than 200 µm. The study carried out this step to eliminate the effect of particle size, which was not the subject of this study [19].

Step 2: Chemical Deproteinization and demineralization

The chemical deproteinization of chitin treatment was performed with 20 wt.% NaOH solution at a solid-to-liquid ratio of 1:20 [7]. This was performed to eliminate the proteins. The mixture was held in 500 mL Erlenmeyer flasks and a stirrer in heating and stirring magnetic equipment for 2 h. After the duration, settling of the mix in ambient conditions was allowed. The chitin particles settled, and the supernatant NaOH decant was washed away with neutral pH water. After chemical deproteinization, removing the minerals to produce chitin was necessary [20]. Chemical demineralization was performed with acid treatment using 6 wt.% hydrochloric acid (HCl). The solid-to-liquid ratio for all processes was set at 1:20, as suggested by [7].

Step 3: Deacetylation

After the deproteinization and demineralization of the raw shell, deacetylation was carried out at various temperatures. Combinations of reaction time, temperature, and NaOH concentrations yield a total of 27 chitosan samples. Each of these samples was characterized with FTIR to determine its DD.

**Figure 1:**
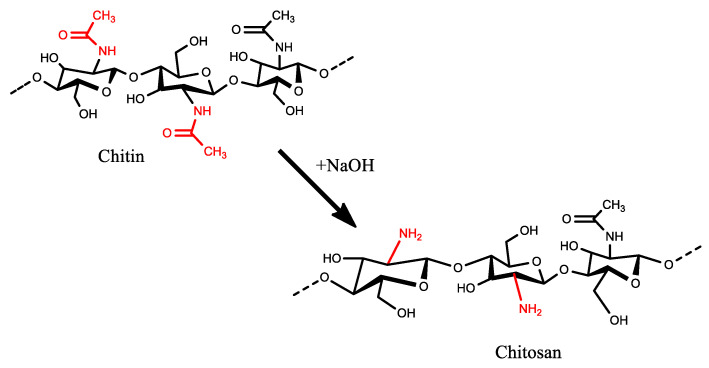
Reaction scheme for chitin deacetylation with NaOH.

Chitin deacetylation encompasses several methods, with the hot alkali treatment being the preferred and extensively utilized approach. This method employs sodium hydroxide (NaOH) in deacetylation (Figure 1).

### 2.4. Optimization Studies

The optimization of chitosan synthesis parameters was inspired by the Box–Behnken design (BBD), and the evaluation of the effect of the independent parameters on DD (response) was evaluated [21]. However, this study did not reduce the runs as suggested by the BBD-coded runs. This was performed to bring insight into the parameters’ interdependencies at specific ranges and establish areas of highest responses, considering the lack of studies that have optimized these conditions. The effect of temperature, reaction time, and NaOH concentration combinations on DD was analyzed. The varied deacetylation process parameters were based on the outcomes of previous studies. Table 1 shows the coded values of the variable conditions (settings) which were varied in Table 2 following all possible combinations. For the analysis of the effect of each parameter, the other two were fixed. The extent of sensitivity of DD to each parameter and combination of parameters was studied. The discussion of DD was further carried out based on the process’s feasibility, with emphasis on reaction time, temperature, and NaOH requirements.

### 2.5. Fabrication of Membranes

The established chitosan of the highest DD was used for the synthesis of the membrane. Firstly, the magnetic stirrer dissolved PES granules in dimethyl sulfoxide (DMSO) solution at room temperature. Once completely dissolved, chitosan was introduced to the mix and left undisturbed for about 24 h to obtain a homogeneous gel (i.e., consistent texture and appearance). PES, chitosan, and DMSO loading were 10, 0.75, and 89 wt.%, respectively. The membrane was then kept in water for 2 h in order for any solvent to be adsorbed from the membrane sheet. The last step was drying the membrane in the oven at 80 °C for 2 h, aiming to remove any water or solvent trapped on the membrane body. This membrane was characterized by FTIR and SEM observations. Similarly, a pristine (pure) PES membrane was fabricated with PES and DMSO loadings of 10 and 90 wt.%, respectively.

### 2.6. Performance Evaluation of Membrane

Performance indication properties of the membranes studied were surface morphology and water contact angle. SEM was used to obtain the surface micrograph of the fabricated pristine PES membrane and the PES/chitosan membrane. The surface micrographs of the two membranes were compared to outline the significant difference in membrane performance. A water contact angle analyzer was used to obtain the contact angle of both membranes. Then, the contact angle was compared to determine the effect of chitosan on hydrophilicity.

## 3. Results and Discussion

### 3.1. Shell and Chitin Characterization

**Figure 2:**
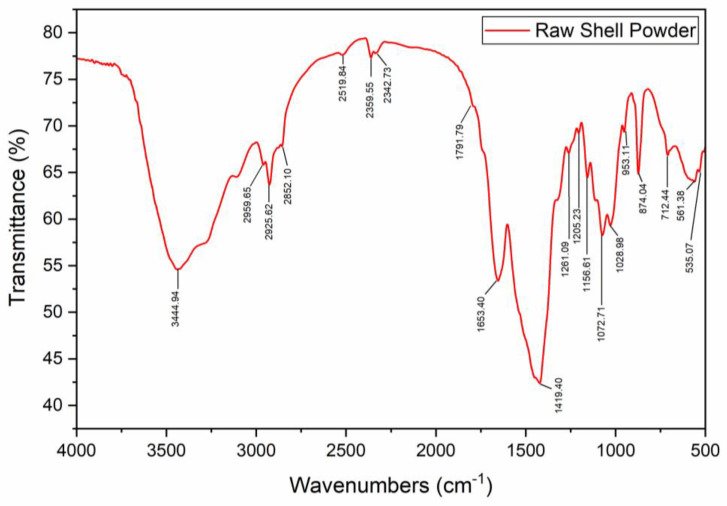
FTIR spectra of raw shell.

Figure 2 shows the FTIR spectra of the raw pulverized shell. The peak at wavenumber 2852 cm^−1^ revealed the presence of the C–H bonds (stretching vibrations) of aliphatic hydrocarbons (alkaline groups). The broad peak at 3441.9 cm^−1^ was attributed to the hydroxyl (O–H) for present water molecules in the shell. Additionally, the broad peaks between 3441 and 2959 cm^−1^ were attributes of the N–H groups. The sharp peak revealed the stretching vibrations for C=O at 1653.4 cm^−1^. The small adsorption band at 2519 cm^−1^ revealed the presence of the carboxylic acid (–COOH) group, located relative to a larger %transmittance value, thus a lower %absorbance, which means a relatively small bond population of this group. The transmittance peaks at 1791.79 and 874.04 cm^−1^ confirm the presence of the calcite, CaCO_3_. The sharp transmittance peak at 1419.40 cm^−1^ was an attribute of the CH_3_ bend and deformation of CH_2_. Similar to the FTIR spectra obtained in this study, [9] studied the properties of structural polysaccharides from shrimp exoskeleton. The results confirmed the presence of C–H (aliphatic), C=O (amine I), O–H, N–H, C–H, –COOH, and calcite groups. These results agree with the ones exhibited in this study. It is also significant to note the material in which these bonds preside within the raw shell. In this regard, ref. [22] reported that raw crustacean shell contains about 75%, 12%, 9%, and 4% of water, proteins, mineral salts, and chitin, respectively, with traces of organic pigments. Based on these results, it was, therefore, valid to presume that O–H and calcite groups are of the water molecule and mineral salts, respectively, but not limited to only these materials.

**Figure 3:**
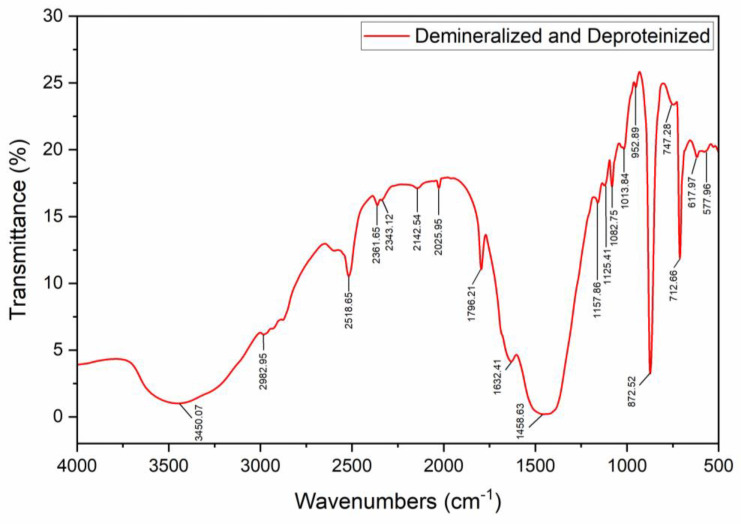
FTIR spectra of chitin.

Figure 3 shows the spectra of the shell after deproteinization and demineralization (chitin). Compared to that of raw shell, the spectra reveal a relatively low transmittance observed between 2700 and 4000 cm^−1^, thus low transmittance of the previously established O–H stretch (of the water molecule) and N–H bonds. This low transmittance indicates a high absorbance, thus a large bond population of these functional groups. The increase in the bond population of the O–H of the water molecule is presumed to be due to comparably insufficient drying, carried out after washing off the NaOH and HCl with de-ionized water. The absence of the sharp peak at approximately 1456 and 874 cm^−1^ suggests the removal of the calcite, CaCO_3_, thus the minerals have been successfully removed from the shell material. Similarly, the absence of the C=O (carboxyl) stretching vibration peak at approximately 1653 cm^−1^ reveals the successful removal of the proteins from which this functional group is found. It is also important to note that deproteinization and demineralization resulted in significant mass losses, as protein and minerals were lost with the discarded NaOH and HCl.

### 3.2. Chitin Yield

Table 3 below shows the yield of chitin produced after the chemical pretreatment (demineralization and deproteination) of shrimp and mussel shells. Generally, the chitin yield differs based on the extraction method used. Recent research has highlighted the potential for production of high chitin yield by alternative methods such as bio-extraction with microorganism-mediated fermentation and biotechnological extraction using lactic acid bacteria and enzymes) [23,24,25]. These methods are both economically friendly and economically viable. Nevertheless, in the current study, common chemical extraction with HCl and NaOH was applied, leading to a chitin yield of 61.4%.

### 3.3. Degree of Deacetylation

Table 4 below shows the %DD (degree of deacetylation) obtained at different temperatures, time, and alkaline concentrations. These three parameters have strong interdependencies such that the trends in DD are not easily observed without further analysis. The standard deviation (CV) values are used to quantify the amount of variation or dispersion in the obtained %DD values. The subsequent section will evaluate the effects of these three parameters on DD, individually and jointly.

**Figure 4:**
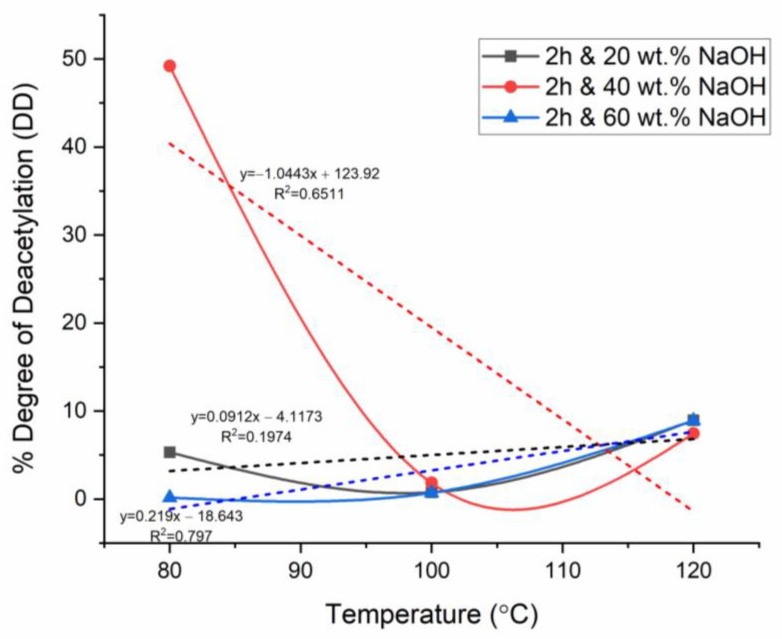
%DD for 2 h deacetylation with variable time and NaOH concentration.

**Figure 5:**
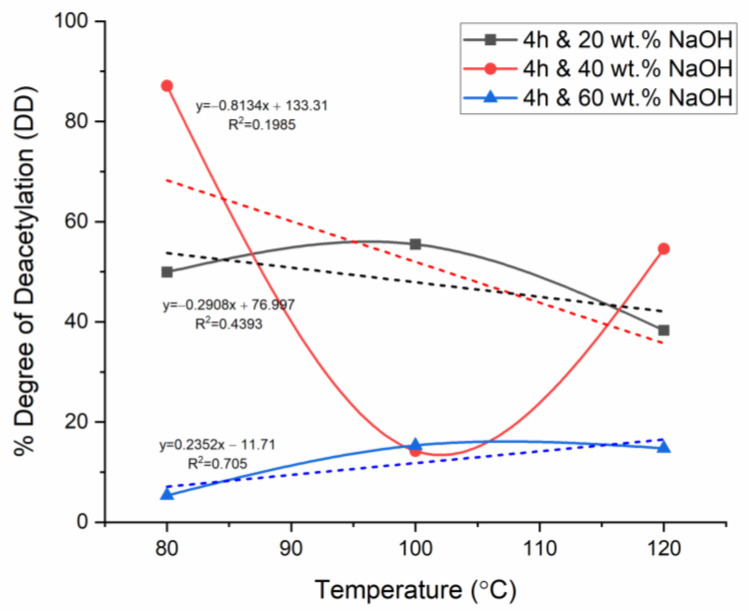
%DD for 4 h deacetylation with variable time and NaOH concentration.

**Figure 6:**
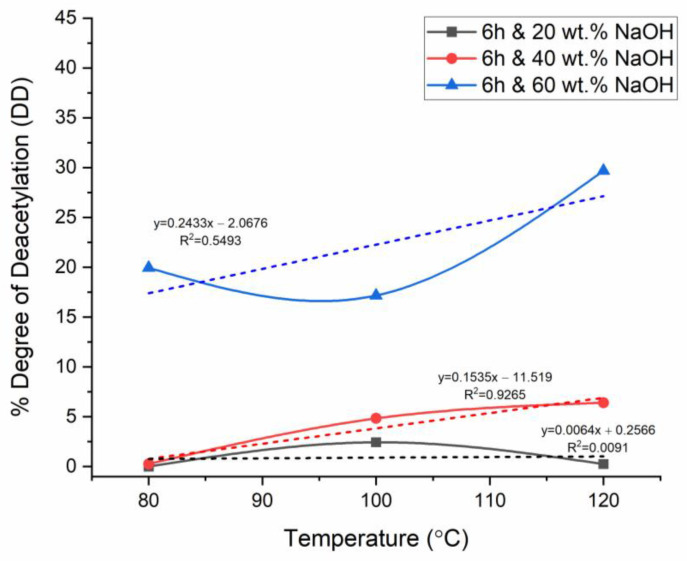
%DD for 6 h deacetylation with variable time and NaOH concentration.

For the analysis of the effect of the parameters on %DD, firstly, the temperature was chosen as the primary independent variable for the plots at fixed reaction times, while the NaOH concentration varied in each figure. This was performed to analyze the effect of temperature and NaOH concentration, whereas the respective figures were compared to investigate the effect of reaction time. The aforementioned comparison method was collated with linear regression analysis with correlation factor (R^2^) values to evaluate the strength of accuracy of the relationship the plots yielded, where R^2^ closer to zero meant poor correlation, thus high uncertainty, whereas R^2^ closer to 1 meant a stronger correlation, thus relatively low uncertainty. In Figure 4, Figure 5 and Figure 6, the dashed lines represent the linear fitting of the curves, with the corresponding linear equations and regression data.

### 3.4. Effect of Time on Chitosan’s Degree of Deacetylation

The effect of time was analyzed by collating and further analyzing the general trends in time in Figure 4, Figure 5 and Figure 6. The results showed that significant deacetylation started at 4 h for the three concentrations of NaOH studied at low temperatures. Fatima [22] investigated the effect of time on chitin deacetylation. The results revealed that the best reaction time is mainly determined by the time it takes to reach the activation energy for deacetylation and break the bond (relative to the temperature and alkaline concentration). In this context, 4 h was determined as the time at which the activation energy was reached, significant deacetylation occurred, and chemical breakdown occurred to release the acetyl groups. Nevertheless, as previously reported in this study, at 4 h of deacetylation, this breakdown of the acetyl group was significant only at a higher NaOH concentration of 40 wt.% NaOH, whereas it was relatively lower at 20 wt.% and 60 wt.% NaOH. Alkaline substrate diffusion on shell substrate increases over time. Fatima [22] further reported the significant interaction between NaOH concentration and reaction time on %DD. Based on this study, the effect of time at lower NaOH and higher NaOH concentrations was analyzed separately. At the lowest NaOH concentration (20 wt.%), the %DD increased between temperatures of 80 and 100 °C for all reaction times explored in this study. When the lowest time (2 h) was used, the extracted product remained predominately as chitin (%DD < 50). The deacetylation was observed to terminate after a time ranging from 4 to 6 h, depending on the NaOH concentration. When 20 wt.% NaOH was used, the deacetylation was incomplete even when the time was increased to a much larger reaction time (6 h). Therefore, for 20 wt.% NaOH, the deacetylation did not significantly occur completely; hence no chitosan was extracted (DD < 50%).

In contrast, when a relatively larger time was used (4–6 h), the %DD showed an exponential decline depending on the NaOH concentration used. Ref. [22] reported the extended reaction time’s effect to be similar to that of higher temperature and to a similar extent. Therefore, keeping the shell surface in contact with an alkaline substrate (NaOH) for longer times above the optimum time (4 h in this study) degraded the deacetylation by reversing the diffusion [7].

### 3.5. Effect of Temperature on Chitosan’s Degree of Deacetylation

The deacetylation of chitin was carried out at temperatures of 80, 100, and 120 °C. This analysis was carried out by comparing Figure 4, Figure 5 and Figure 6 observations Analysis of the %DD measurement results at 4 h of deacetylation showed a %DD increase of approximately 10% (49.94–55.50%) at a low concentration of 20 wt.% NaOH and a thermal increase of 20 °C (80–100 °C). This %DD variation seems less significant than that at the same conditions but with higher NaOH concentration (60 wt.%), where %DD increases by approximately 65% (5.35–15.33%). This discrepancy could be an error in the result analysis. However, this increase in %DD is crucial with NaOH increase from 20 to 40 wt.%. This is because when the NaOH concentration was increased from 20 to 40 wt.%, the %DD saw a decrease of 83.67% (87.11–14.22). In this stage, the temperature was a variable that inhibited the acetyl groups’ ability to bond and accelerate the deacetylation reaction. Ref. [26] studied the kinetics of N-deacetylation of chitin extracted from shrimp shells. The results showed that %DD values increase with temperature and NaOH concentration as the reaction time increases. This is in support of what was observed in the current study. The increase in %DD was attributed to the structural change in chitin at high temperatures, as confirmed by FTIR studies. Furthermore, the obtained results confirmed that the interaction between temperature (thermal energy) and concertation is the main criteria to take into account for the deacetylation reaction.

### 3.6. Effect of NaOH Concentration on Chitosan’s Degree of Deacetylation

This study explored deacetylation at 20, 40, and 60 wt.% NaOH at varied temperatures and reaction times. This analysis was carried out by comparing Figure 4, Figure 5 and Figure 6. When deacetylation was carried out at a low alkaline concentration (20 wt.%), the %DD did not exceed 50%; thus, no chitosan was produced, and the product remained predominantly chitin. This was the case regardless of the temperature and reaction time used. This study established the deacetylation temperature and NaOH concentration to be the key factors that affect the deacetylation at a fixed time. At low concentrations of NaOH (20 wt.%), the deacetylation time became higher with improving %DD at temperatures below optimum. This showed that the %DD significantly depends on the NaOH concentration due to the inaccessible acetamide groups in the polymer chain. Ref. [27] studied the demineralization of crustacean shells in preparation for chitin, and ref. [28] characterized shrimp shell deprotonation; the observation made from these studies revealed that the behavior of %DD’s dependence on NaOH concentration is mainly explained by N-deacetylation of chitin takes place at chitin’s level of amorphous region and further passes from edge to the crystalline region’s interior. Various studies have reported the reaction’s equilibrium and chitosan degradation as the limiting deacetylation factor [7].

In the present study, NaOH was increased from 20 to 40 wt.% NaOH when the time and temperature were fixed at 4 h and 80 °C, respectively. The %DD saw an increase of 42.67% (49.94 to 87.11); this was only applicable to the temperature of 80 °C as previously reported that an increase in temperature with the same concentration increase would result in a collective effect that would degrade the %DD. Therefore, it is necessary to report that the joint effect of temperature and NaOH concentration is the most significant compared to the effect of reaction time, provided that the chosen time allows for the activation energy of deacetylation to be reached (optimum time). Ref. [7] evaluated the performance of PES/chitosan on acid mine drainage (AMD) treatment; the study involved chitin deacetylation at varied conditions to establish the temperature and NaOH concentration yielding a high %DD at a fixed reaction time of 6 h. The assumption postulated in the literature was that 6 h of deacetylation is sufficient for significant deacetylation. In contrast, as revealed in the current study, the time alone is not a major factor in %DD determination, provided the chosen time is sufficient for the activation energy to be achieved. Ref. [22] reported that as little as 60 min is enough for significant deacetylation to occur, depending on the NaOH concentration and temperature used. This meant that reaction time could be fixed, and NaOH concentration and reaction temperature could be adjusted/varied for the already established reaction time.

Ref. [10] obtained the highest %DD of 95.97% at 6 h, 100 °C and 40 wt.% NaOH whereas in the current study, the highest %DD of 87.11% was obtained at 4 h, 80 °C, and 40 wt.% NaOH. Based on the discussed effect of the three parameters, the compared conditions should yield comparable %DD values. Nevertheless, the standard deviation accounting for the accuracy in the study by [10] was 0.82, whereas in the current study, it was 1.30; thus, the current study’s %DD value had low certainty compared to the former. However, the current and the referenced study used FTIR band ratios A1320 and A1340 for %DD calculations with different formulas. The current study used Equation (1), and the study in [10] used Equation (2) below. The formulas each hold their tolerance for inaccuracy based on their method derivation assumptions and would yield precise but not necessarily equal %DD value.

(1) $$\%DA=13.9\left(A1320/A1420\right)-12.2$$

(2) $$\%DA=31.92\left(A1320/A1420\right)-12.56$$

This study also reported the degradation of %DD at 120 °C regardless of other conditions, indicating that the effect of temperature at high temperatures becomes independent and degrades deacetylation regardless of other conditions. The current study revealed 4 h as the appropriate time for significant deacetylation and 80 °C as the optimized temperature. This is relatively feasible for application as it requires less thermal energy input than 100 and 120 °C. The NaOH concentration of 40 wt.% yields a relatively lower %DD of 87.11%; therefore, it is considered the main parameter for optimization with a basis on the previously reported interactions between the shell surface and NaOH substrate.

As previously reported, the NaOH concentration and the deacetylation are the main factors to consider for effective deacetylation, provided that the reaction time is sufficient to achieve activation energy under the used conditions. The reaction time was established as 4 h, and the temperature (thermal energy) was established as 80 °C. Ref. [22] reported that high temperatures cause chitosan degradation (%DD reduces), causing a drop in viscosity and molecular weight, thus affecting the solubility of chitosan. In contrast, NaOH concentration does not affect molecular weight. Therefore, it is established that the optimization of chitosan synthesis parameters should significantly focus on the minimum reaction time necessary to achieve activation energy, the minimum temperature required to provide sufficient thermal energy without the risk of chitosan degradation, and NaOH concentration optimized for temperature and reaction time parameters. A study by [22] reported that establishing the NaOH concentration on the basis of reagent diffusion mechanism, reaction rate, kinetics, and activation energy would make it easier to establish feasible and significant deacetylation. In the same study, chitin deacetylation was reported to follow pseudo-first-order kinetics for all temperatures between 25 and 120 °C. This would provide a new and simplified basis for deacetylation optimization.

This study used the conditions that yielded the 87.11%DD to synthesize chitosan from chitin for infusion in fabricating the PES/chitosan membrane, whose performance properties were further evaluated.

### 3.7. Quality Evaluation of PES Membranes vs. PES/Chitosan Membrane

Previous research has demonstrated that the presence of chitosan in PES membranes significantly improves their separation properties. For instance, in a study by [10], adding chitosan improved the membrane’s hydrophilicity and water flux. Moreover, it has also led to enhanced antifouling properties and increased rejection rates for various solutes such as dyes and heavy metal ions [29,30]. In this study, the fabrication of membranes followed loadings of casting gel suggested by [10]. Two membranes were synthesized for comparison: the pristine PES membrane and the PES/chitosan membrane. This aimed at comparing the performance-indicating characteristics of the two membranes. The PES membranes were prepared with 10% and 90% polyethersulphone (PES) and dimethyl sulfoxide (DMSO), respectively. At the same time, the PES/chitosan membrane was fabricated with casting gel comprising 10, 0.75, and 89.25 wt.% of PES, chitosan, and DMSO, respectively (thus PES/0.75 chitosan membrane).

### 3.8. Physiochemical Characterization of Pristine PES and PES/0.75 Chitosan Membranes

**Figure 7:**
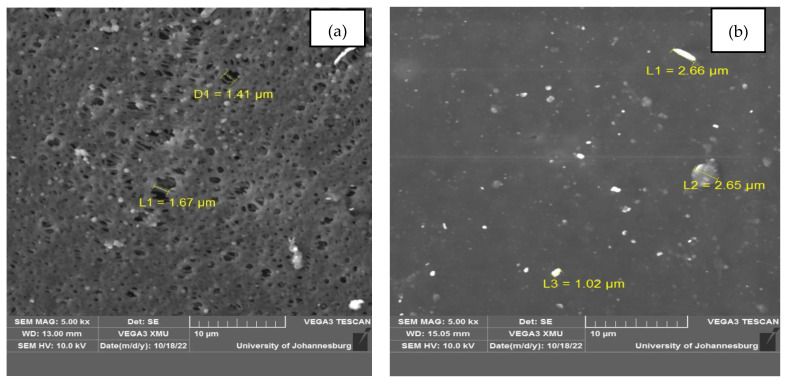
Surface morphology of (**a**) pristine PES and (**b**) PES/0.75 chitosan membranes.

Figure 7a,b depicts the surface morphology of pristine PES and PES/0.75 chitosan membranes, respectively. Both SEM micrographs revealed the uniform porous structure of a typical ultrafiltration (UF) membrane. A noticeable difference was observed between the two membranes, such that the roughness character of the membrane reduced with chitosan infusion, making the PES/0.75 chitosan membrane smoother than the pristine PES membrane. Ref. [10] reported similar results and concluded that it was due to chitosan forming complexes with material structures and filling empty spaces on the membrane’s layer. Adding chitosan particles reduced the pore sizes of the membrane.

Nevertheless, it is significant to report that the material observed with sizes of 2.66 and 2.65 µm on the membrane’s layer is chitosan particles; this correlated with the particle size distribution of the chitosan. Ref. [8] evaluated the surface morphology of pristine PES and tannin iron complex PES(TA-FeIII) membranes; the results revealed that the viscosity of the casting solution was increased by the addition of the complex, thus affecting the pore size. In the context of the current study, the pore size of the PES/0.75 chitosan membrane could have been affected by adding chitosan. Nevertheless, in the context of AMD treatment, the large pore sizes of the pristine PES membrane only serve the benefit of reducing pore plugging by particulate material. However, it also results in penetration of saline water and consequent failure of AMD treatment [31]. Hence, the PES/0.75 chitosan membrane would presumably serve better contaminant rejection and permeate flux than the pristine PES membrane.

### 3.9. Membrane Hydrophilicity (Water Contact Angle)

**Figure 8:**
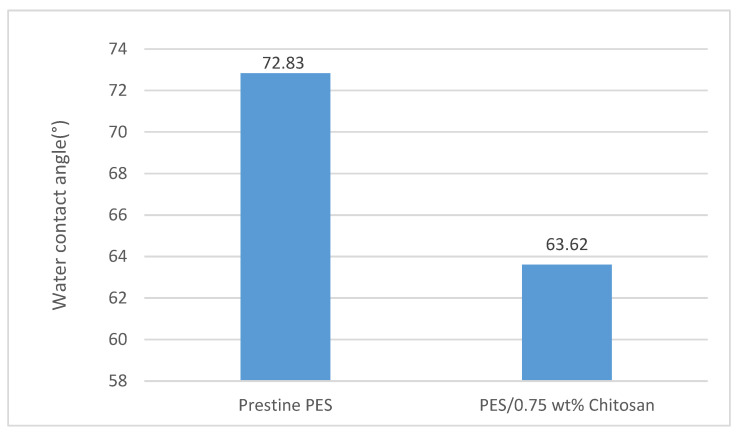
Contact angle for pristine PES and PES/0.75 chitosan membranes.

The contact angle between the water droplets and the surfaces of the membranes was used to evaluate the hydrophilicity of the membranes. The contact angle indicates flux and hydrophilicity behavior; hence, it is considered a vital membrane characteristic. Figure 8 shows the bar graphs comparing the water contact angle of the two fabricated membranes.

As observed in Figure 8, the water contact angle of pristine PES and PES/0.75 chitosan membranes had water contact angles of 72.83° and 63.62°, respectively. A lower contact angle corresponds to higher hydrophilicity; thus, the PES/0.75 chitosan has higher surface hydrophilicity than the pristine PES membrane. Ref. [8] studied the surface hydrophilicity of pristine PES membrane; the results revealed a contact angle of 75°. This value is only +2.89% different from the water contact angle obtained in the current study (72.83°), thus, validating the strong accuracy of the contact angle obtained in the present study. Similarly, ref. [7] studied the hydrophilicity of PES/0.75 chitosan (95.6%DD) membrane; the results revealed 61° for contact angle. This contact angle value is only −4.11% different from the one obtained in the current study. With a difference range of ±5% (error tolerance), the findings are strongly precise, thus confirming the accuracy or certainty of the contact angle results obtained in the current study. It is also significant to outline that the %DD of chitosan used in this study was 87.11%, whereas the authors of [7] utilized 96%. The difference in the contact angles is accounted for by the difference between the %DD values used in the respective studies; thus, with low %DD, there is a limited number of amide and amide groups on the membrane’s matrix and surface, which has been reported to aid with water transport.

Mathaba [7] further reported that high chitosan %DD improves the hydrophilicity and porosity of membranes. The higher the hydrophilicity, the more improved the contaminant rejection and permeate flux [10]. In the context of acid mine drainage (AMD), the PES/0.75 chitosan membrane would presumably have higher permeated flux and contaminant rejection due to improved water transportation and hydrophilicity. Nevertheless, these enhancement effects improve with the increase in %DD of chitosan. It is also essential to report that increasing the chitosan loading would increase water adsorption by the membrane, thus reducing the adsorption of pollutants on the membrane. Hence, the chitosan loading utilized in this study was the optimum one (0.75% chitosan) established by [7].

### 3.10. Techno-Economic Discussion of the Established Optimum Conditions

The techno-economic analysis is fundamentally a cost-profit comparison of different engineering alternatives [32]. It is also significant to assess the techno-economic analysis of the established optimum conditions for chitosan synthesis; 80 °C, 40 wt.% NaOH and 4 h of deacetylation. The production of chitosan is recognized for its high demand for water and reactants, primarily due to the necessity for a substantial liquid-to-solid ratio (i.e., 1:20) during the contact and reaction processes. The most employed method of chitosan synthesis is hot alkaline treatment, where NaOH is most preferred due to its unique properties. Studies have shown that the first step for cost reduction would depend on the effective recycling of the effluents. Hence, ref. [33] proposed recycling deacetylation and extraction solutions as an alternative to a more cost-effective process. Ref. [33] evaluated the techno-economic analysis of chemical deacetylation of chitin at 90 °C, 50 wt.% NaOH (solids to liquid, 1:24), and a mean DD of 89% was obtained at these conditions. The values of the factors used in [33] are significantly higher than those used in the current study (87%DD), with no significant difference in DD. Further, ref. [33] simulated the chitosan synthesis process to assess its feasibility at an industrial scale, with a profit margin of results revealed that the process was profitable and cost-effective. Generally, the chemical chitin deacetylation process can be further improved with applications of water optimization techniques, as suggested by [34]. Using chitin, sourced from crustacean shells such as shrimp, presents an opportunity for cost-effective raw material in chitosan production. Given that a significant portion of these shells constitutes waste from the seafood and fish industry, this approach leads to significant cost reduction and improved economic viability for the chitosan manufacturing process [35].

Ref. [36] evaluated the microwave deacetylation of chitin, the highest DD of 82.8% was obtained at 40% of NaOH solution for 12 min, working with 650 W. Compared to the current study, the deacetylation time is significantly reduced. Although the energy requirement in microwave deacetylation appears significantly higher, it outweighs the longer deacetylation time in chemical deacetylation [37]. Nevertheless, at an industrial scale, microwave chitin deacetylation becomes a challenging process, and further research is needed for process optimization and assessment of its economic viability [36]. Considering the current state of chitosan synthesis, the optimum conditions established in this study are merited as energy efficient and economically viable relative to the currently explored methods.

### 3.11. Challenges in the Implementation of PES/Chitosan Membranes

The implementation of PES/chitosan membranes presents several challenges that need to be addressed for optimal performance. The synthesis process involves the use of high concentrations of NaOH and requires temperatures of 120 °C for prolonged periods, leading to high energy demands, thus high operational costs [10]. While these implications improve the performance of the PES, membrane, their utilization at a large scale raises environmental and safety concerns. The use of high concentrations of NaOH in the synthesis process significantly influences the properties of PES/chitosan membranes, as shown by [10]. Recent research has highlighted the importance of taking environmental and safety factors into account when fabricating PES/chitosan membranes. One study by [38] stressed the need to minimize chitosan membrane manufacturing’s impact on the environment, especially in CO_2_ separation processes, while improving permeability. Meanwhile, the authors of [39,40] explored different ways of leveraging chitosan for enhanced performance: Ref. [40] looked at how varying polyethylene glycol compositions affected oily wastewater treatment efficacy via chitosan membranes, while ref. [39] investigated using a waste-derived additive from golden snail shells to improve antifouling properties in PES-based water purification systems. Additionally, Mathaba’s [10] findings suggested that increasing deacetylation degrees could boost acid mine drainage filtration efficiency with PES-chitosan materials—together demonstrating significant potential for utilizing this biopolymer compound towards bettering overall sustainability within these discordant fields.

The use of large amount of acidic and alkaline materials generally induces environmental and safety concerns in any engineering setting, hence recent developments in membrane technology have concentrated on enhancing sustainability and reducing environmental impact. Scholars such as Mohamed [41] and Kim [42] emphasize the utilization of eco-friendly solvents and techniques to decrease waste during membrane production. Meanwhile, Echarri [38] assesses the ecological repercussions of chitosan membranes, stressing the need for improving their permeability. Shi’s [43] research points out that biopolymer-based membranes like chitosan hold immense potential in mitigating environmental issues. Taken together, these studies reiterate how incorporating sustainable practices while fabricating PES/chitosan membranes is crucial towards a successful implementation process.

## 4. Conclusions

In this study, the highest chitosan degree of deacetylation (%DD) achieved was 87.11% at 80 °C, using 40 wt.% NaOH for 4 h during chitin deacetylation. Further optimization could enhance %DD by exploring NaOH concentrations between 40 wt.% and 60 wt.%. The %DD was more sensitive to temperature and NaOH concentration combinations than reaction time at a low concentration of 20 wt.%, %DD increased by approximately 10% (from 49.94% to 55.50%) with a thermal increase of 20 °C (from 80 °C to 100 °C). However, this variation was less significant than conditions with higher NaOH concentration (60 wt.%), where %DD increased by approximately 65%. The optimal conditions for significant chitin deacetylation are 80 °C for 4 h with 40 wt.% NaOH, which is economically viable compared to current commercial methods. The PES/0.75 chitosan membrane (87.11%DD) exhibited improved surface hydrophilicity (63.62° water contact angle) compared to the pristine PES membrane (72.83° water contact angle), suggesting better membrane performance. Future research should explore NaOH concentrations that yield %DD higher than 87.11% at 4 h and 80 °C of deacetylation. Measure pH during deproteinization wash-off (targeting pH 6–7) to reduce the impact of residual proteins. Characterize chitosan using at least two methods (e.g., FTIR and pH-metric/conductometric/UV) for accurate %DD assessment. Use two adsorption band ratio pairs (A1655/A3450 and A1320/A1420) for precision. Additionally, conduct a study optimizing NaOH concentration based on reagent diffusion, reaction rate, kinetics, and activation energy in chitin deacetylation. Lastly, it is recommended that future research should include a pH of zero-point charge (pHzpc) for chitosan synthesized at different conditions. Lastly, the fabrication of PES/chitosan membranes involves high energy requirements and the use of strong acids and alkaline materials for extended periods, leading to high operational costs and raising environmental and safety concerns. Therefore, future research should prioritize the exploration of eco-friendly alternatives in the fabrication of these membranes.

## Figures and Tables

**Table 1:** Independent variables and their coded values.

Level	Temperature (°C)	Time (h)	NaOH Concentration (wt.%)
−1	80	2	20
0	100	4	40
1	120	6	60

**Table 2:** Chitosan synthesis process conditions.

Sample Number	Temperature (°C)	Time (h)	NaOH (wt.%)
1	80	2	20
2	80	2	40
3	80	2	60
4	80	4	20
5	80	4	40
6	80	4	60
7	80	6	20
8	80	6	40
9	80	6	60
10	100	2	20
11	100	2	40
12	100	2	60
13	100	4	20
14	100	4	40
15	100	4	60
16	100	6	20
17	100	6	40
18	100	6	60
19	120	2	20
20	120	2	40
21	120	2	60
22	120	4	20
23	120	4	40
24	120	4	60
25	120	6	20
26	120	6	40
27	120	6	60

**Table 3:** Chitin yield.

Initial Dry Mass (g)	Chitin Mass (g)	%Yield
117.86	72.864	61.4

**Table 4:** Obtained DD at different deacetylation conditions.

Sample Number	Temperature (°C)	Time (h)	NaOH (wt.%)	%DD	Standard Deviation, CV
1	80	2	20	5.30	0.04
2	80	2	40	49.20	0.17
3	80	2	60	0.16	0.01
4	80	4	20	49.94	0.44
5	80	4	40	87.11	1.30
6	80	4	60	5.35	0.03
7	80	6	20	407.97	32.05
8	80	6	40	0.26	0.02
9	80	6	60	19.94	0.12
10	100	2	20	0.75	0.07
11	100	2	40	1.83	0.08
12	100	2	60	0.71	0.00
13	100	4	20	55.50	0.36
14	100	4	40	14.22	0.04
15	100	4	60	15.33	0.04
16	100	6	20	2.43	0.06
17	100	6	40	4.83	0.04
18	100	6	60	17.17	0.05
19	120	2	20	8.94	0.08
20	120	2	40	7.43	0.05
21	120	2	60	8.92	0.04
22	120	4	20	38.30	0.16
23	120	4	40	54.58	0.55
24	120	4	60	14.76	0.06
25	120	6	20	0.26	0.04
26	120	6	40	6.41	0.06
27	120	6	60	29.67	0.13

## Data Availability

Data are contained within the article.
